# The Possible Non-Mutational Causes of FVIII Deficiency: Non-Coding RNAs and Acquired Hemophilia A

**DOI:** 10.3389/fmed.2021.654197

**Published:** 2021-04-15

**Authors:** Alina-Andreea Zimta, Ionut Hotea, Melen Brinza, Cristina Blag, Sabina Iluta, Catalin Constantinescu, Atamyrat Bashimov, Elisabeth-Antonia Marchis-Hund, Alexandra Coudsy, Laetitia Muller-Mohnssen, Noemi Dirzu, Diana Gulei, Delia Dima, Margit Serban, Daniel Coriu, Ciprian Tomuleasa

**Affiliations:** ^1^Medfuture Research Center for Advanced Medicine, Iuliu Hatieganu University of Medicine and Pharmacy, Cluj Napoca, Romania; ^2^Department of Hematology, Iuliu Hatieganu University of Medicine and Pharmacy, Cluj Napoca, Romania; ^3^Department of Hematology, Ion Chiricuta Clinical Cancer Center, Cluj Napoca, Romania; ^4^Department of Hematology, Fundeni Clinical Institute, Bucharest, Romania; ^5^Department of Hematology, Carol Davila University of Medicine and Pharmacy, Bucharest, Romania; ^6^Department of Pediatrics, Iuliu Hatieganu University of Medicine and Pharmacy, Cluj Napoca, Romania; ^7^Department of Hematology, Emergency Clinical Children's Hospital, Cluj Napoca, Romania; ^8^Intensive Care Unit, Clinical Hospital for Infectious Diseases, Cluj Napoca, Romania; ^9^Louis Turcanu Emergency Children's Hospital, Timisoara, Romania; ^10^European Hemophilia Treatment Center, Timisoara, Romania; ^11^Department of Pediatrics, Victor Babes University of Medicine and Pharmacy, Timisoara, Romania

**Keywords:** hemophilia, non-coding RNAs, epigenetics, hypothesis, acquired bleeding disorder

## Abstract

Hemophilia type A (HA) is the most common type of blood coagulation disorder. While the vast majority of cases are inherited and caused by mutations in the *F8* gene, recent data raises new questions regarding the non-heritability of this disease, as well as how other molecular mechanisms might lead to the development of HA or increase the severity of the disease. Some data suggest that miRNAs may affect the severity of HA, but for some patients, miRNA-based interference might cause HA, in the absence of an *F8* mutation. A mechanism in HA installation that is also worth investigating and which could be identified in the future is the epigenetic silencing of the *F8* gene that might be only temporarily. Acquired HA is increasingly reported and as more cases are identified, the description of the disease might become challenging, as cases without FVIII autoantibodies might be identified.

## Introduction—“Classical” View of Hemophilia Type A and the Still Unanswered Questions

Hemophilia type A (HA) is a blood coagulation disorder described as an inherited condition caused by mutations in the *F8* gene. Two mutations are found the most often in HA: intron 22 inversion (inv 22) and intron 1 inversion. Inv 22 is the most frequent mutation with different reported frequency, depending on the population: 35% (1), 45% of severe HA cases (2). Inv1 is the second most common mutation of HA in severe HA, with a different reported frequency of between 5 (3) and 7% (1). One large populational study from the US, the My Life, Our Future (MLOF) project concluded that the inv22 is found in 42% of severe HA cases and 3.7% of mild/moderate cases. Inv1 was found in 1.2% of severe HA cases and 0.2% of mild/moderate HA cases. In the majority of HA cases, which account for around 79.5% of patients, the most common type of mutation is the missense mutation (4).

Every year, new mutations in the *F8* gene are discovered and the connection with severity of the disease (5–7) is reevaluated to gain more insight into HA and the reasons for different degrees of severity. Even though this continuing approach sustains patient stratification and risk evaluation, it does not offer a complete picture in terms of recent discoveries of FVIII repression at a transcriptional or translational level independent of the type of *F8* mutation. More proof regarding the non-mutational influence of the circulating level of FVIII has been gathered from studies on healthy individuals, in whom FVIII levels increase with age (8).

Hematology presents these scenarios by analyzing the non-genetic influence of HA onset and severity. These may be related to the epigenetic repression of the *F8* gene, non-coding RNA post-translational inhibition of the *F8* mRNA, or direct repression of the FVIII protein. Through these analyses, new types of HA might be identified and monitored differently than in the case of “classical” HA.

## MiRNAs Effects on Severity, Bleeding, and Complications—THE Need to Introduce More ncRNA Analysis in the Context of HA

The first line of evidence regarding the non-genetic causes of HA onset and progression comes from non-coding RNAs (ncRNAs), especially microRNAs (miRNAs). These RNAs can interact with the *F8* mRNA and suppress its translation.

The evidence so far is scarce and done on a small number of patients, but it points to some missing details in the current overall view of HA. Jankowska et al. analyzed the miRNA able to bind to the *F8* 3′UTR through *in silico* data analysis and through RNA-affinity purification. The data was then compared to RNAseq results from 2 mild/moderate HA patients without *F8* mutation and one severe HA patient with no known *F8* mutation. In total, 22 up-regulated and 8 down-regulated miRNAs were found to be common with the miRNA targeting the *F8* gene. MiR-19b-3p, miR-186-5p, miR-30c-5p, miR-93-3p, and miR-1246, all of which target *F8* are also up-regulated in HA, and their expression increases with the severity of HA. At the same time, miR-93-5p, miR-196b-5p, let-7g-5p, miR-320a, and miR-221-3p showed inverse correlation with HA severity. The miR-19b-3p and miR-186-5p were experimentally demonstrated to target and down-regulate the *F8* gene (9).

Through *in silico* analysis, Rosset et al. concluded that miR-26a-5p and miR-26b-5p have a higher energetic score for binding to a mutated form of *F8* mRNA (the c.8728 A>G variant) than in the case of the WT *F8* gene. The *F8* mutations are not disease-associated (10). Even if this data still needs experimental confirmation, it might show that even in the case of HA patients with known *F8* mutation the microRNA completes the picture of this pathology and its severity by influencing the level of *F8* gene variants, with no association with HA disease.

Throughout hemophilia progression, miRNAs also reflect acute episodes. It was proven that in hemophilia-induced arthropathy, the pro-inflammatory NF-kB pathway is activated and leads to the production of the pro-inflammatory cytokines interleukin (IL)-1β, IL-6, and tumor necrosis factor α (TNF α). MiRNAs that activate the NF-kB pathway, such as miR-155, miR-9, miR-16, and miR-181b, were also up-regulated. In the hemophilic joints, there is an increase in pro-angiogenic factors, such as the vascular endothelial growth factor α (VEGFα), hypoxia-inducible factor 1α (HIF1α), and matrix metalloproteinases (MMPs), that degrade the chondrocyte matrix. The miRNAs related to apoptosis were also mostly up-regulated, as is the case of miR-155, miR-186, miR-23a (11). In another study, published by Sen and Jayandharan in India, miR-15b expression was reported to specifically drop during a bleeding episode, as well as in the following 6 weeks. In an animal model, they later confirmed that the local induction of miR-15b overexpression leads to down-regulation of pro-angiogenic factors VEGF-α and HIF-2α, together with a reduction in the MMPs responsible for chondral degeneration (MMP1, MMP3, MMP9, and MMP14) (12). These results clearly show that miRNAs are capable of influencing HA progression in the context of different genetic backgrounds, thus the value of RNA analysis even in HA with known mutations might better aid the correct formulation of treatment. Pipe et al. considered missense mutations within the B domain, which were reported in patients with HA. They explored whether the B domain is dispensable for the secretion and function of *F8*, and hypothesized that these mutations should not cause HA in these patients. Plasmid vectors containing B domain missense mutations are reported to be linked to moderate and severe HA and have been analyzed for their effect on synthesis and secretion compared with *F8* wild-type (WT) following transient transfection into COS-1 and CHO cells *in vitro*. The mutants were then expressed *in vivo* in a HA mouse model by hydrodynamic tail-vein injection. *F8* activity and the antigen levels for all mutants expressed into the conditioned media of COS-1 and CHO cells were similar to FV8 WT. The plasma expression of these mutants was similar to *F8* WT in HA mice. They concluded that most missense mutations within the FVIII B domain would be unlikely to lead to severe HA and that the majority of such missense mutations represent polymorphisms or non-pathologic mutations (13).

The RNASeq technology has proven its applications in identifying the molecular basis for allogenic inhibitor development in HA, analysis made from peripheral blood. The analysis reported that in the contexts of allogenic antibody development, there is an increase in the expression of inflammatory initiating molecules, namely NLRP3 and TLR8 (14). The next generation sequencing (NGS) of intronic regions describes several mechanisms for the non-coding RNA post-translational inhibition of *F8* mRNA or direct repression of the FVIII protein, including the creation of splice sites resulting in the incorporation of intronic sequences into the mRNA and intronic mutations at the exon-intron boundaries, resulting in the excision of exons or deep intronic mutations occurring anywhere in the gene. This leads to sequences within the mRNA that can, for example, insert stop codons, resulting in premature truncations. Using NGS, Bach et al. identified 23 deep intronic candidate variants in several *F8* introns, including six recurrent variants and three variants previously described. In each of the 15 patients analyzed, at least one deep intronic variant in the *F8* gene was described to predict the alteration of *F8* mRNA splicing. Reduced *F8* mRNA levels or stability would thus be compatible with the patients' mild to moderate HA phenotypes (15). The same group also presented mRNA analysis of novel *F8* mutations with possible impact on splicing in four HA patients with silent mutations and seven patients with intronic variants close to or within splice site consensus sequences. Seven of the 11 mutations examined *in vitro* were shown to affect *F8* mRNA splicing and the results were compared to *in silico* predictions (16). Castaman et al. aimed to analyze *F8* mRNA for mutations in five families with mild HA with no apparent genomic mutation and a reduced response to desmopressin. They concluded that even if rare, deep intronic variations may be responsible for mild HA where no other *F8* mutations are identified and may be associated with a reduced biologic response to desmopressin (17). At the University of Bonn, Pezeshkpoor et al. determined *F8* and vWF von Willebrand factor (vWF) activity and antigen levels and performed vWF-*F8* binding and vWF-collagen binding assays, as well as vWF multimer analysis. vWF was completely sequenced to exclude mutations. The *F8* locus, including the introns, was sequenced using overlapping long-range PCRs combined with an NGS approach. Moreover, the *F8* mRNA was analyzed quantitatively and qualitatively by real-time PCR and overlapping reverse transcription PCRs, respectively (18). By systematically excluding all possible causes of HA, they concluded that deep intronic mutations in *F8*, although rare, cause abnormal mRNA splicing, leading to mild HA.

## miRNAs in the Prevention of Successful Gene Therapy in HA and How to Prevent Their Effect

Another way in which ncRNAs interfere in HA progression might be linked to their impairment of the efficacy for future treatments. Evidence of this comes from hemophilia type B (HB) but is still highly valuable in understanding the principle. Gene therapy is becoming a successful strategy for both HA and HB treatment. It is more efficient for HB according to clinical trials, which is why more evidence comes from HB (19–21). However, the therapy is often suppressed by the targeted inhibition of exogenous *F8* or *F9* mRNA through miRNAs (22–24). MiRNAs and other types of non-coding RNAs, such as small interfering RNAs (siRNAs), which target a factor from the coagulation cascade can impair the application of gene therapy for HA or HB. Other miRNAs or siRNAs have a therapeutic role in these pathologies. For instance, Fitusiran is a siRNA targeting antithrombin (AT) that increases the level of thrombin and increases blood coagulability in HA or HB (25). Some studies have proposed and successfully implemented the inclusion of miRNA target sites on the lentiviral vector, that would act as a miRNA-sponge. One such example was the use of the miR-142-3p sponge sequence in the lentiviral vectors, also containing the F9 gene. The vector transfected the extravascular hematopoietic lineage and managed to successfully induce FIX expression (26). In HA, this strategy was not as successful in the first trial as for HB. Besides the *F8* gene, the vector contained a liver-restricted promoter and GP54 envelope glycoprotein and the target sites for miR-142. The expression of FVIII was restricted to the liver. Thus, more pronounced results were obtained in the case of transfection in mice neonates, as opposed to adults. This occurred because neonates did not have a fully developed immune system and the initial transfected cells expanded naturally throughout their lifetime (27).

Nourse et al. investigated the miRNA-mediated regulation of the hemostatic system and sed an integrative screening approach that combines functional aspects of miRNA silencing with biophysical miRNA interaction based on RNA pull-downs coupled to NGS. By assessing a panel of 27 hemostasis-associated gene 3'UTRs, they showed that the majority had substantial Dicer-dependent silencing capability, suggesting functional miRNA targeting. They identified 150 specific miRNA interactions with 14 3'UTRs, of which 52, involving 40 miRNAs, were functionally confirmed. This includes cooperative miRNA regulation of key hemostatic genes comprising procoagulant (*F7, F8, F11, FGA, FGG*, and *KLKB1*) and anticoagulant (*SERPINA10, PROZ, SERPIND1*, and *SERPINC1*) as well as fibrinolytic (PLG) components (28). In the Netherlands, Vossen et al. described 3'UTR variants in coagulation genes that influence coagulation factor levels and venous thrombosis risk. The 3'UTR of coagulation genes were sequenced in subjects with extremely high or low plasma levels of these factors in two case-control studies. MiR-544 caused a decrease of the luciferase activity not observed with an rs4253430 mutated vector. Thus, they stated that microRNAs are candidates that play a role both in hemostasis and thrombosis (29).

We believe that the non-specificity of microRNAs leads to these contradictory reports, since miRNA targets hundreds of other genes besides *F8* mRNA, which might also contribute to the results. It could be helpful to look at different species of ncRNAs, some of which might be more specific. This could be done with the RNA Seq technique by gathering a number of patients and comparing them with healthy individuals, HA patients with *F8* mutations, and HA patients without *F8* mutation. This is a critically important point, and a key challenge that affected the studies mentioned previously, in investigating an ultra-rare subpopulation within a rare disease.

There are two long non-coding RNAs (lncRNAs), specifically NONHSAT139215 and NONHSAT139219, that are located in the *F8* gene loci. Through molecular docking simulation of the interaction between the 3D structure of the aforementioned lncRNAs and the FVIII protein, it was proven that these two interact. Upon comparative RT-PCR analysis of the expression of these two ncRNAs, it was proven that they have a decreased level in HA patients compared to healthy controls. The severity of the decrease in expression was also correlated with the severity of hemophilia (30). MiR-1246 is markedly increased in HA patients compared to healthy controls, regardless of the co-present mutations in the *F8* gene and its expression is high also in patients who have not developed anti-FVIII inhibitors. This microRNA binds in the 931–937 of *F8* 3′UTR (31).

## NcRNAs as the Sole Cause of HA in the Absence of *F8* Mutations-Possibly a New Type of HA

Besides the above-mentioned examples, the rare cases where the *F8* gene is wild-type and there are no autoantibodies, but still the patients develop HA, are interesting uncommon scenarios for these rare bleeding disorders. Although there are some instances in the study of these cases, more detailed research is required as they may constitute a new type of HA that is non-inherited and that is different from acquired hemophilia, meaning the absence of auto-antibodies.

More precisely, it has been reported that 2–5% of HA patients do not have any mutations in the *F8* gene, but still, the FVIII protein is absent from the blood and HA develops. Jankowska et al., from the United States Food and Drug Administration, did a miRNA Seq analysis on two cases of mild/moderate HA patients, with WT *F8* gene, compared with healthy donors. This was a significant step forward from the genetic-orientated analysis of hemophilia toward a transcriptomic orientated one. From the list of miRNAs up-regulated in HA patients, miR-374b-5p and miR-30c-5b was also found to directly target *F8* gene mRNAs and thus impair FVIII protein translation, according to *in silico* analysis (microRNA.org, TargetScan, miRDB) and an experimental validation done on HEK293T, HeLa, and MS1 cells. Moreover, miR-30c inhibition up-regulates the level of FVIII (9, 32). This was a huge change in perspective as classical HA can no longer be predicted in a family solely based on *F8* mutation status and other mechanisms that are still being uncovered can lead to the development of HA.

As Salvajer and Pipe argue, the results from miRNA studies in *F8* WT HA cases are contradictory and the definitive association between certain miRNAs and *F8* mRNA levels is still too early to be assessed. They discuss another study analyzing miRNAs that targeted genes from the coagulation cascade that did not find the miR-374b-5p and miR-30c-5b as targets of the *F8* gene. More detailed analysis on a larger cohort of HA patients with unmutated *F8* gene needs to be conducted (33).

## The Epigenetic Influence of HA Severity and a Possible Connection With HA Development in Some Cases

Besides the significant role of ncRNAs in inducing HA and influencing its severity, a lesser-known mechanism is the epigenetic silencing of the *F8* gene, which in theory might be reversible. Proof of this influence comes from how existing mutations affect the epigenetic status of the *F8* gene. The well-known inversions in the intron 1 or intron 22 of the *F8* gene affect the methylation pattern of the surrounding sites (CpG island methylation), regardless of other confounding effects, such as age. Epigenetic changes can differentiate between intron 1 or intron 22 inversion (34). The role of epigenetics is revealed by the heritability of mutations in the *F8* gene. An analysis of different members of the same family, all carriers of Xq28 deletions, which included the *F8* gene, showed different degrees of severity of the disease. For instance, some members showed preferential epigenetic inactivation of the X chromosome with Xq28 deletion, while others did not show inactivation of the mutated X chromosome (35). The potential link between epigenetics and HA severity must be confirmed by further thorough studies as there is uncertainty regarding the methylation pattern of the *F8* gene. Zimmermann et al. reported no difference in the methylation pattern of hemophilic patients and the controls (36). Genomic inversions in the *F8* (Xq28) region are associated with detectable changes in methylation levels, thus inv1 and inv22 were associated with changes in the methylation pattern of CpG regions. However, this only applies in *F8* inversions. Considering that the HA is caused by a great variety of mutations in the *F8* genes, the epigenetic silencing of the *F8* gene may be mutation specific and carry some *F8* variants, and epigenetic disease-specific epigenetic changes might be completely absent.

## AHA—A Recently Described HA With a Non-Mutational Background With Insufficiently Understood Epigenetic Influences

**Figure 1:**
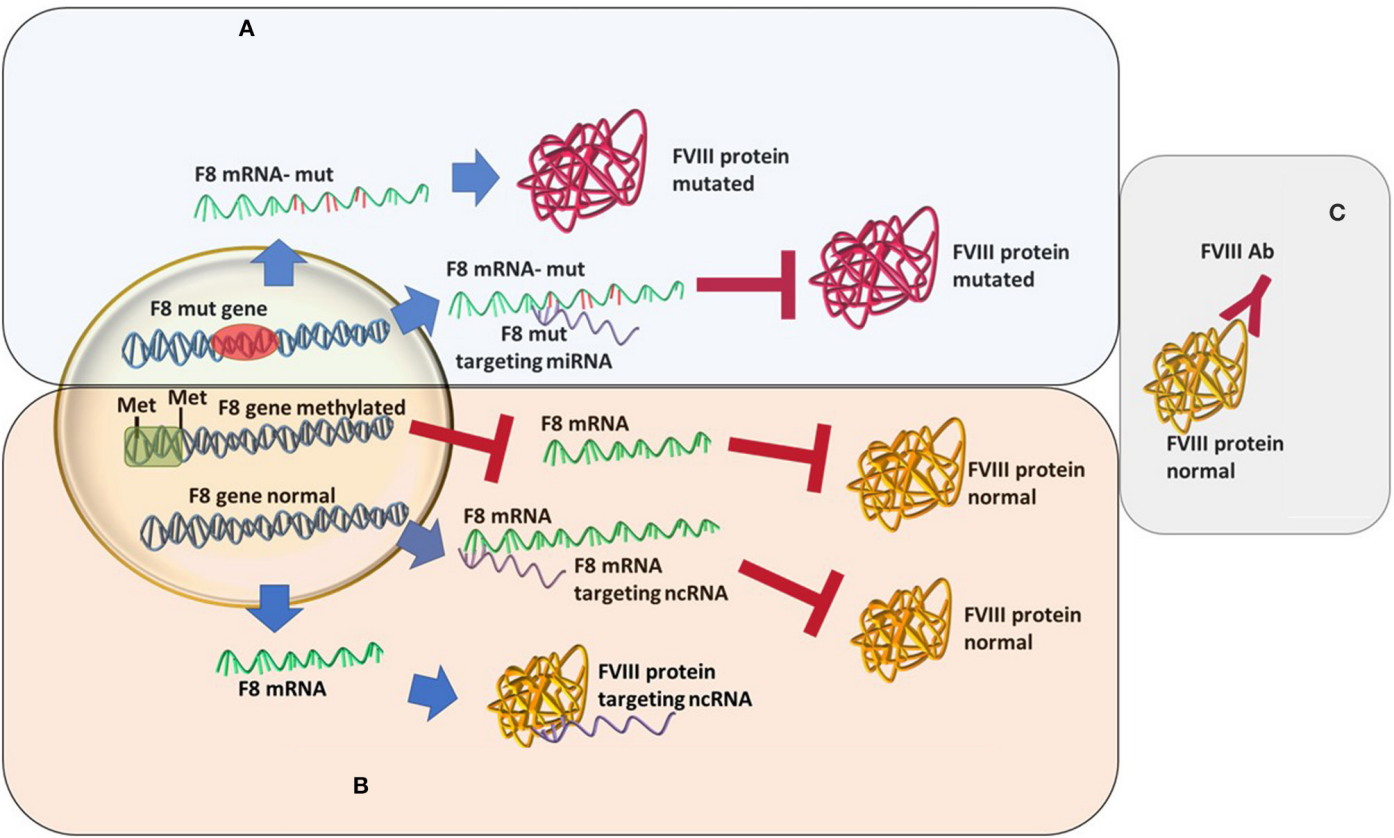
Different types of hemophilia type A and how epigenetic silencing of ncRNA repression might influence the FVIII level regardless of the mutation status. **(A)** Classic hemophilia-new mutations are found, miRNAs changes in expression increases severity. **(B)** Non-inherented rare hemophilia-still unclear or lesser known molecular mechanisms. **(C)** Acquired hemophilia-recently characterized.

An increasingly well-known cause of HA that differs from the “classical” view is the recently described acquired hemophilia (AHA). AHA is a very rare, acquired bleeding disorder caused by *F8* autoantibodies, which neutralize *F8* activity. These inhibitors differ from alloantibodies against *F8*, which can occur in congenital HA after repeated exposure to plasma-derived or recombinant FVIII products. In most cases, the disease occurs suddenly in subjects without a personal or familiar history of bleeding, with symptoms that may be mild, moderate, or severe. AHA mostly affects female patients while HA is mainly caused by a mutation in *F8* and affects male patients (37–39). The bleeding pattern of AHA is different from that of HA. Most patients with *F8* autoantibodies have hemorrhages in the skin, muscles, or soft tissues and mucous membranes, whereas hemarthrosis, a typical feature of congenital *F8* deficiency, is uncommon. This disease develops during a person's lifetime, usually in special conditions, such as pregnancy, after major surgery or very often linked to underlying malignancy, and it is described as an autoimmune disease (40, 41). In acquired hemophilia, the newly developed antibodies are different from the alloantibodies developed as a response to r*F8* treatment in congenital HA (39) (Figure 1). These represent an acute response with more severe consequences. The disease may be idiopathic or present with severe symptoms. The paradigm of acquired hemophilia has also shifted over the last few years. It was shown that in two acquired hemophilia patients a point mutation was found (c.8899G>A) in the *F8* gene (42). As follows, more evidence may come if acquired HA patients will also go through an analysis of the ncRNA or epigenetic mark-up.

Non-mutation induced HA is defined as a sudden abnormal hemorrhage diagnosed in a patient, not on anticoagulation, without personal or family bleeding history, with an isolated prolonged aPTT and a mixing study consistent with an inhibitor. The abnormal prolongation of aPTT before surgery must always be investigated. Still, for 30% of cases, only laboratory alterations occur. A diagnosis must be confirmed by a hematology department with expertise in coagulation disorders, as well as in the management of inhibitors against coagulation factors. Clinical management recommends avoiding invasive procedures. If necessary, procedures must be performed in a hemophilia center or after consultation with it. Physicians are advised to investigate the underlying cause of the disease as soon as the diagnosis of acquired HA (AHA) has been made. Should the underlying cause be identified, therapy should be initiated.

The many medical conditions that trigger AHA, such as autoimmune disorders, malignancy, drug reaction, or even idiopathic, can affect the patient to such extent that they may require complex observation or treatment in a High-Dependency Unit (HDU) or the intensive care unit (ICU) (43, 44). To correctly manage such a patient before admission and during the first minutes, doctors should follow a focused and straightforward approach. The complications that in the end lead to the death of the patient are those of a delayed diagnosis (mostly because of a lack of experience), difficulty achieving hemostasis, other associated diseases that further decompensate and lead to organ failure and complications of treatment (such as venous thrombosis). The clinical problems that these patients pose are: how do we control the bleeding and how do we get rid of the autoantibodies. The ABC approach is the most commonly used worldwide, but during hemorrhage situations, the correct one is CABC (45). Before assessing the airway, try to find the source of bleeding (if there is an evident one) and apply hemostasis, if it is possible. Continue with the airway (A) and breathing (B) check, where it is mandatory to maintain a permeable airway and give oxygen if the SpO_2_ is <94% in atmospheric air, to fully saturate the hemoglobin remaining in the circulation and achieve optimal delivery of oxygen to the tissues. Returning to the (C)-circulation step, adequate intravenous access [at least two 18 gauge (G) intravenous catheters] are available and if a shock state is suspected, one should begin fluid infusion (crystalloid or colloid) or blood-derived products. During bleeding situations make sure the patient is connected to a monitor where there is a constant evaluation of clinical signs, such as ECG, SpO_2_, and blood pressure. Daily clinical examination and laboratory studies are mandatory. Pay attention to the need for invasive procedures and avoidance of antiplatelet, anticoagulant, or other therapies that may cause further bleeding. Most patients present with bleeding symptoms and require the initiation of antihemorrhagic treatment. By knowing the antibodies titers the correct medication may be chosen. If the inhibitor titer is ≤ 5 BU human FVIII concentrates or DDAVP can be used as first-line treatment. For the cases with high-titer (>5 BU), bypassing agents can be used, such as activated prothrombin complex concentrate (aPCC), which contains activated FII, FVII, FIX, and FX (dependent vitamin-K factors) and recombinant activated FVII (rFVIIa), achieving hemostasis by generating thrombin (in the absence of FVIII) at the site of bleeding (46). The concomitant use of both agents is not recommended, only in life-threatening situations, where hemostasis could not be achieved by the use of one agent (47). In the situations of mucosal bleeding, an antifibrinolytic drug can be used (epsilon-aminocaproic acid or tranexamic acid) (48). After the hemostasis has been achieved, ongoing treatment should be continued (as prophylaxis) to prevent recurrence of bleeding. Furthermore, the treatment is oriented on the possibility of elimination of the antibodies. The recommended first-line treatment for autoantibody eradication is an immunosuppressive regimen mainly consisting of prednisone (1 mg/kg/day) alone or in combination with oral cyclophosphamide (50–100 mg/day), with the target being an undetectable inhibitor (<0.6 BU) and normal FVIII levels (>50%) (49). Rituximab, calcineurin inhibitors cyclosporine, tacrolimus) and mycophenolate mofetil are alternatives to rituximab in patients who do not respond to first-line treatment (50–52).

At times, because the management of these patients is complex, and there is difficulty in achieving hemostasis, liaison with hemophilia specialists and treatment centers is highly recommended.

## Conclusion

HA is a disease far more complex than initially thought. In the future, there might be more varieties of the disease than originally thought, as more studies focus on the epigenetic and transcriptomic background of the disease. These might not only explain the differences in the severity of the disease but also offer answers to the cases with no mutations and no autoantibodies. Based on the research literature and critical analysis, we propose that there are at least two more types of uncharacterized HA: the epigenetic one and one caused by ncRNA. Thus, future research should focus on deciphering the role of both epigenetic events and non-coding RNAs in the pathophysiology of both “classical HA,” as well as AHA.

## Author Contributions

All authors contributed in the design and writing of the manuscript.

## Conflict of Interest

The authors declare that the research was conducted in the absence of any commercial or financial relationships that could be construed as a potential conflict of interest.
